# Chimeric antigen receptor T cells targeting the GM3(Neu5Gc) ganglioside

**DOI:** 10.3389/fimmu.2024.1331345

**Published:** 2024-02-02

**Authors:** Julia Heinzelbecker, Marte Fauskanger, Ida Jonson, Ute Krengel, Geir Åge Løset, Ludvig Munthe, Anders Tveita

**Affiliations:** ^1^Department of Immunology and Transfusion Medicine, Oslo University Hospital, Oslo, Norway; ^2^K.G. Jebsen Centre for B cell malignancies, University of Oslo, Oslo, Norway; ^3^Department of Chemistry, Faculty of Mathematics and Natural Sciences, University of Oslo, Oslo, Norway; ^4^Department of Biosciences, Faculty of Mathematics and Natural Sciences University of Oslo, Oslo, Norway; ^5^Nextera AS, Oslo, Norway

**Keywords:** CAR (chimeric antigen receptor) T cells, GM3(Neu5Gc) ganglioside, carbohydrate antigen, solid tumor, immunotherapy, 14F7, antibody

## Abstract

Chimeric antigen receptor (CAR) T cell technology has ushered in a new era of immunotherapy, enabling the targeting of a broad range of surface antigens, surpassing the limitations of traditional T cell epitopes. Despite the wide range of non-protein tumor-associated antigens, the advancement in crafting CAR T cells for these targets has been limited. Owing to an evolutionary defect in the CMP-Neu5Ac hydroxylase (CMAH) that abolishes the synthesis of CMP-Neu5Gc from CMP-Neu5Ac, Neu5Gc is generally absent in human tissues. Despite this, Neu5Gc-containing antigens, including the ganglioside GM3(Neu5Gc) have consistently been observed on tumor cells across a variety of human malignancies. This restricted expression makes GM3(Neu5Gc) an appealing and highly specific target for immunotherapy. In this study, we designed and evaluated 14F7-28z CAR T cells, with a targeting unit derived from the GM3(Neu5Gc)-specific murine antibody 14F7. These cells exhibited exceptional specificity, proficiently targeting GM3(Neu5Gc)-expressing murine tumor cells in syngeneic mouse models, ranging from B cell malignancies to epithelial tumors, without compromising safety. Notably, human tumor cells enhanced with murine *Cmah* were effectively targeted and eliminated by the 14F7 CAR T cells. Nonetheless, despite the detectable presence of GM3(Neu5Gc) in unmodified human tumor xenografts, the levels were insufficient to trigger a tumoricidal T-cell response with the current CAR T cell configuration. Overall, our findings highlight the potential of targeting the GM3(Neu5Gc) ganglioside using CAR T cells across a variety of cancers and set the stage for the optimization of 14F7-based therapies for future human clinical application.

## Introduction

Alterations in glycosylation patterns of malignant cells represents a hallmark of cancer, and modified glycans present a promising source of novel tumor-associated antigens. Notably, the GD2 ganglioside and the Tn glycoform of the membrane phosphoprotein MUC1 have been probed as potential CAR T cell therapy targets, with both demonstrating promising outcomes in animal models of solid tumors (1, 2). Still, the repertoire of CAR T cell strategies targeting non-protein antigens is limited.

The *N*-glycolyl-monosialodihexosyl ganglioside GM3(Neu5Gc) has garnered attention as a potential immunotherapeutic target, given its selective expression across a spectrum of tumor cells, including adenocarcinomas (breast and colon), non-small cell lung cancer, melanoma, lymphomas, and retinoblastomas (3–9). This expression profile is supported by immunohistochemistry studies (3–8) and *in vivo* scintigraphy using the GM3(Neu5Gc)-specific antibody 14F7 (9), which showed minimal expression in healthy tissues. Recently, the molecular recognition mechanism of 14F7 has been elucidated using protein engineering (10, 11), X-ray crystallography (12–14) and liposome binding studies (15), and cytotoxic effects of the 14F7 mAb have been comprehensively studied (6, 16–18).

**Figure 1:**
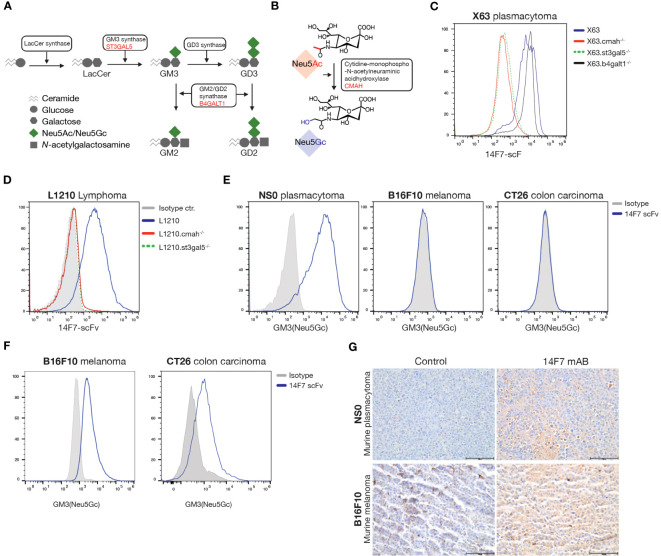
GM3(Neu5Gc) synthesis and expression in murine cell lines. **(A)** Schematic overview of the ganglioside biosynthesis pathway. Genes names encoding the enzymes responsible for conversion steps are given in red. Created with BioRender. **(B)** Drawing illustrating the hydroxylation of *N*-acetylneuraminic acid (Neu5Ac) to *N*-glycolylneuraminic acid (Neu5Gc), catalyzed by cytidine monophospho-*N*-acetylneuraminic acid hydroxylase (CMAH). **(C, D)** Histograms showing surface 14F7 scFv staining in wild type (WT) and *Cmah* -, *St3gal5*-, and *B4galt1* knock-out variants of the murine plasmacytoma cell line X63 and the B lymphoma cell line L1210. **(E)** Histograms showing 14F7 scFv staining of murine cell lines under standard cell culturing conditions. **(F)** Histograms showing 14F7 scFv staining of murine colon cancer CT26 and melanoma B16F10 (B16) cells *ex vivo* isolated from established s.c. tumors (>5mm diameter). **(G)** 14F7 mAb immunohistochemistry staining of established NS0 and B16 tumors (>5mm diameter). Scale bars indicate 100 µm.

The biosynthesis of GM3 involves the enzyme ST3GAL5, which transfers sialic acid from cytidine 5′-monophosphate-sialic acid (CMP-SA) to its precursor lactosylceramide (LacCer) (illustrated in **Figure 1A**). Depending on the specific sialic acid used, either *N*-acetylneuraminic acid (Neu5Ac) or *N*-glycolylneuraminic acid (Neu5Gc), the resultant products are GM3(Neu5Ac) or GM3(Neu5Gc), respectively. The generation of Neu5Gc hinges on the enzyme CMP-Neu5Ac hydroxylase (CMAH), which facilitates the conversion of CMP-Neu5Ac to CMP-Neu5Gc (depicted in **Figure 1B**). Intriguingly, due to a 92-bp deletion in the human *CMAH* gene, Neu5Gc is absent in humans under normal conditions (19). Yet, Neu5Gc-containing glycoproteins and glycolipids, including GM3(Neu5Gc) have been identified on human tumor cells (20).

There is accumulating evidence that Neu5Gc, when ingested, can be integrated into human cells (20–26). While the precise mechanism for its preferential accumulation in malignant cells remains elusive, it has been suggested that cellular hypoxia might promote the acquisition of Neu5Gc, potentially driven by the upregulation of the sialic acid transport protein, sialin (SLC17A5), under hypoxic conditions (27).

A recent study using 14F7-derived mouse/human VH/VL scFvs chimeras indicated that intratumoral administration of anti-GM3(Neu5Gc) CAR T cells can delay tumor outgrowth in m*Cmah*-expressing ovarian cancer xenografts, but not in mice challenged with wild-type cancer cells (28). In our current investigation, we designed GM3(Neu5Gc)-targeted CAR T cells based on the murine 14F7 mAb to explore their therapeutic potential of 14F7-28z CAR T cells against established tumors of both murine and human origins. To this end, we utilized an extensively characterized murine scFv fragment (14F7c1) (12, 13, 15) derived by phage display-based light chain shuffling of the original 14F7 mAb (10). Our data reveal that while 14F7-28z CAR T cells injected intravenously have potent effects against established murine tumors, the expression levels of GM3(Neu5Gc) in human tumor xenografts might be suboptimal for effective CAR T-mediated targeting in its current form.

## Results

### 14F7 mAb specifically recognizes GM3(Neu5Gc)

Unlike humans, most mammals, including mice, possess a functional *Cmah* gene. Previous studies have shown that the 14F7 mAb binds abundantly to the cell surface of the murine myeloma cell line P3X63Ag8.653 (X63) (16). To validate the binding specificity of 14F7, we used CRISPR/Cas9 to ablate *Cmah* in X63 cells, which led to a complete loss of 14F7 binding (**Figure 1C**). Introducing nonsense mutations in the gene for the ganglioside GM3 synthase lactosylceramide α-2,3-sialyltransferase (*St3gal5* (**Figure 1A**), also resulted in a loss of binding (**Figure 1C**). However, the elimination of β-1,4-galactosyltransferase (*B4galt1*), which converts sialic acid precursors to the related GM2(Neu5Gc) ganglioside, did not impact binding (**Figure 1C**). Similar observations were made for L1210 B-cell lymphoma and NS0 plasmacytoma cells (**Figure 1D** and **Supplementary Figure 1A**). These findings align with prior data, emphasizing the specificity of 14F7 for Neu5Gc-containing GM3 gangliosides and its lack of cross-reactivity with other closely related Neu5Gc-containing gangliosides (3).

Next, we assessed various murine tumor cells for GM3(Neu5Gc) surface expression. Most cell types, including B16F10 melanoma and CT26 colon carcinoma cells, showed no detectable 14F7 staining under standard *in vitro* culturing conditions (**Figure 1E** and **Supplementary Table 1**). Contrary to B-cell-derived cell lines X63, NS0, and L1210, most murine cell lines reportedly express negligible amounts of the CMAH enzyme *in vitro* (29). Accordingly, the sialylation of GM3 under *in vitro* conditions predominantly results in GM3(Neu5Ac) (29). However, subcutaneous tumors from mice with B16 and CT26 cell lines exhibited strong GM3(Neu5Gc) surface expression, as determined by flow cytometry and immunohistochemistry (**Figures 1F, G**). This suggests that these tumor cell lines either accumulate the ganglioside externally or upregulate the CMAH enzyme *in vivo.*

### Murine 14F7 CAR T cells efficiently eliminate GM3(Neu5Gc)-expressing tumor cells *in vitro*

**Figure 2:**
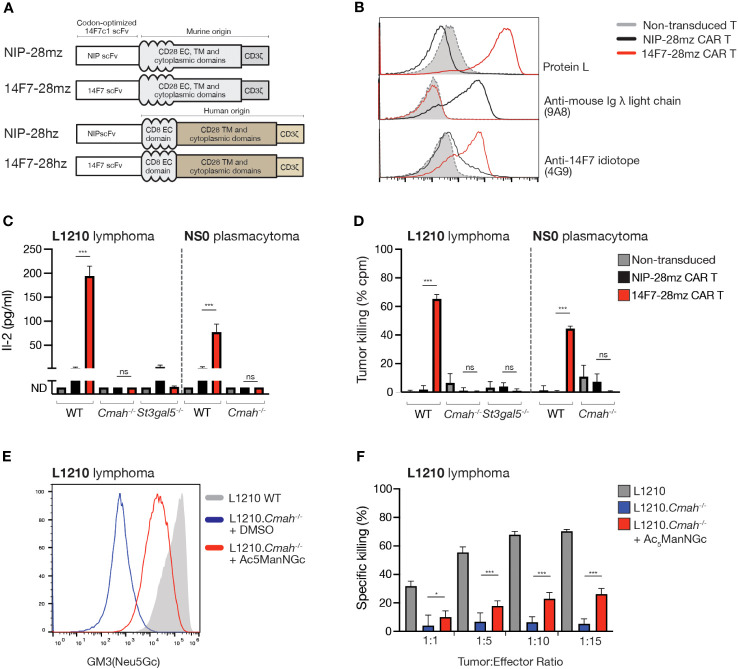
14F7-28mz CAR T cells specifically recognize and eliminate GM3(Neu5Gc) expressing murine cell lines. **(A)** Schematic design of murine and human versions of CAR targeting 14F7 (14F7-28mz and 14F7-28hz) and irrelevant controls targeting the hapten 4-hydroxy-3-iodo-5-nitrophenylacetic acid (NIP; NIP-28hz and NIP-28hz). **(B)** Flow cytometry characterization of 14F7-targeted (14F7-28mz) and control (NIP-28zm) CAR T cells by surface staining with recombinant protein L, anti-mouse lambda light chain mAb (9A8), and an anti-14F7 idiotope mAb (4G9). Staining of non-transduced murine T cells is shown in grey. **(C)** IL-2 concentration in culture medium following 24h co-incubation of CAR T cells with L1210, L1210.*Cmah*^-/-^, and L1210.*St3gal5*^-/-^ or NS0 cells. Shown is one representative experiment out of two, expressed as mean ± SD. **(D)** *In vitro* killing of L1210, L1210.*Cmah*^-/-^ and L1210.*St3gal5*^-/-^ or NS0 cells following 24h co-incubation with 14F7-28mz CAR T cells. Shown is one representative experiment of three (6-8 replicates per group, mean ± SD). **(E)** Histogram showing staining with 14F7 scFv of L1210 and L1210.*Cmah*^-/-^ lymphoma cells under normal cell culturing conditions and L1210.*Cmah*^-/-^ cells metabolically labeled with a sialic acid precursor *N*-Glycolyl-d-mannosamine pentaacetate (Ac_5_ManNGc) (vehicle=DMSO). **(F)** *In vitro* killing of L1210, L1210.*Cmah*^-/-^ and L1210.*Cmah*^-/-^ cells metabolically labeled with 10 µM Ac_5_ManNGc following 24h co-culture with CAR T cells. Shown is one representative experiment of two (n=6-8 per group, mean ± SD). * p<0.05; *** p<0.001; ns= not significant (p> 0.05)

We constructed a 14F7-containing CAR with a murine CD28 transmembrane/signaling domain and a CD3 zeta signaling domain (14F7-28mz) (**Figure 2A**). As a non-targeted negative control, we generated a CAR targeting the hapten 4-hydroxy-3-iodo-5-nitrophenylacetate (NIP; NIP-28mz), which is not expressed in murine cells. Using recombinant protein L, we detected 14F7-28mz CAR expression on primary murine T cells, which retained binding to the 14F7 anti-idiotype antibody 4G9 (**Figure 2B**). Murine CD8^+^ 14F7-28mz CAR T cells were selectively activated when co-cultured with L1210 cells (**Figure 2C**). They efficiently killed wild-type L1210 cells, but not L1210.*Cmah*^-/-^ or L1210.*St3gal5*^-/-^ cells (**Figure 2C**), confirming *in vitro* functionality and specificity. Similar outcomes were observed with NS0 (**Figures 2C, D**) and X63 cells (*data not shown*). As L1210.*Cmah*^-/-^ cells cannot synthesize endogenous Neu5Gc from Neu5Ac, we explored their ability to incorporate Neu5Gc from external sources. Bovine serum used in cell culture contains millimolar amounts of Neu5Gc in fetal bovine serum (FBS) (30), and previous studies have suggested that this may serve as a source of GM3(Neu5Gc) expression in cells cultured in FBS-containing medium (26, 28). In our hands, despite extensive testing, no detectable 14F7 binding (by mAb or scFv staining) or 14F7-28mz CAR T-mediated killing of L1210.*Cmah*^-/-^ cells was observed when cultured in medium supplemented with up to 30% heat-inactivated or non-heat-inactivated fetal bovine serum (**Supplementary Figure 1B** and data not shown). However, when cultured with the cell-permeable Neu5Gc sialic acid precursor *N*-glycolyl-d-mannosamine (Ac_5_ManNGc), L1210.*Cmah*^-/-^ cells displayed detectable GM3(Neu5Gc) surface expression after several days (**Figure 2E**). These metabolically labeled cells were selectively eliminated by 14F7-28mz CAR T cells (**Figure 2F**), suggesting that metabolic labeling can allow sufficient GM3(Neu5Gc) accumulation for CAR T cell recognition and elimination.

### Murine 14F7 CAR T cells efficiently eliminate large established tumors

**Figure 3:**
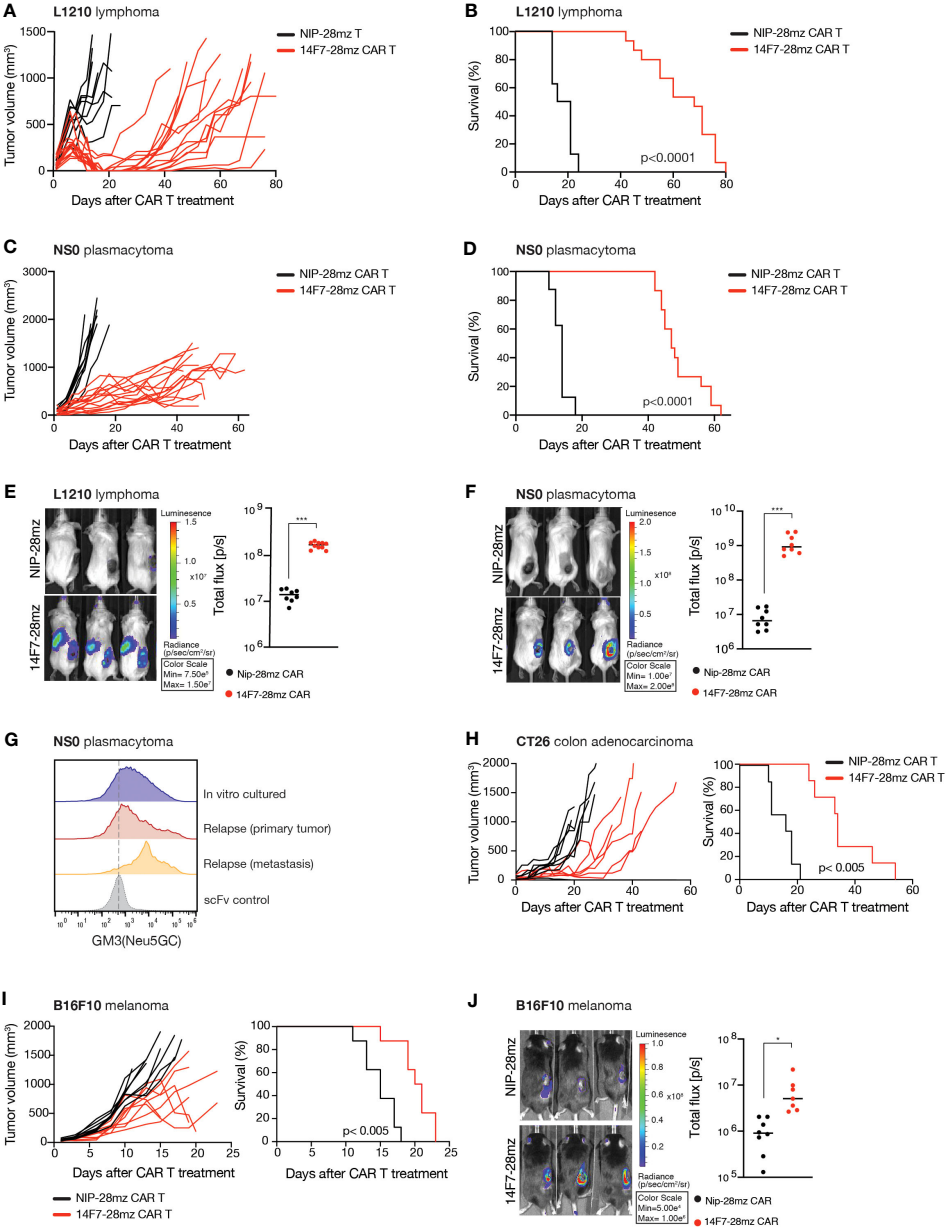
Adoptive transfer of 14F7-28mz significantly delays tumor growth and prolongs survival in several murine tumor models. Survival and tumor volume following adoptive transfer of Neu5Gc-targeting CAR T cells (14F7-28mz) or non-targeted CAR T (NIP-28mz) in mice harboring established (>5mm) subcutaneous tumors. X-axis shows days after T cell transfer. T cells were transferred on day +7 **(A-D, G, H)** or day +10 **(I)** after tumor injection. **(A, B)** Survival and tumor volume measurements following adoptive transfer of 14F7-28mz or NIP-28mz CAR T cells in mice harboring established (>5mm) s.c. L1210 lymphomas (n=8-12) or **(C, D)** s.c NS0 plasmacytomas (n=8-12). **(E)** Representative bioluminescence imaging (left panel) showing accumulation of 14F7-28mz CAR T cell at the tumor side and quantification of whole-body bioluminescence signal (right panel) on day 7 after adoptive transfer for both the L1210 tumor model **(F)** and the NS0 tumor model (n=8) **(G)**. Flow cytometry histograms showing 14F7 scFv staining of cancer cells grown *in vitro* or *ex vivo* isolated from s.c. NS0 primary tumor site and a regional lymph node metastasis following relapse after CAR T therapy. Lower line shows staining with an irrelevant scFv. **(H)** Survival and tumor volume measurements following adoptive transfer of 14F7-28mz or non-targeted CAR T in mice harboring established (>5mm) s.c. CT26 colon cancer (n=7-8) **(I)** or i.d. B16 melanoma tumors (n=8). **(J)** Representative bioluminescence imaging (left panel) showing accumulation of 14F7-28mz CAR T cell localized to the B16 tumor side and quantification of whole-body bioluminescence signal (right panel) on day 7 after adoptive transfer (n=8). * p<0.05; *** p<0.001.

In mice, the functional *Cmah* gene allows GM3(Neu5Gc) expression in healthy tissue, raising potential concerns about on-target off-tumor toxicity. To address this and to verify 14F7-28mz functionality *in vivo*, we tested 14F7-28mz CAR T therapy in Rag1^-/-^ BALB/c mice with large, established subcutaneous L1210 tumors. Treatment with a single i.v. injection of CAR T cells consistently reduced tumor size and delayed tumor growth, enhancing survival compared to NIP-28mz CAR T-treated controls (**Figures 3A, B**). Daily monitoring showed no significant weight loss or other side effects from the CAR T cells. Similar outcomes were observed with 14F7-28mz CAR T in mice with established NS0 plasmacytomas (**Figures 3C, D**). Bioluminescence imaging of luciferase-expressing CAR T cells confirmed selective homing to the tumor site for both L1210 (**Figure 3E**) and NS0 (**Figure 3F**) experiments. No evidence of antigen loss in tumor cells was observed after relapse, with 14F7 staining levels comparable to *in vitro* cultured cells (**Figure 3G**). Moreover, cells from relapsed tumors remained susceptible to *in vitro* killing by 14F7 CAR T cells (*data not shown*).

Given that *in vitro* GM3(Neu5Gc)-negative tumor cell lines, B16 melanoma (C57Bl6) and CT26 colon cancer (BALB/c), showed 14F7 binding during *in vivo* growth, we tested 14F7-28mz CAR T therapy in immunocompetent mice with syngeneic solid tumors. The adoptive transfer of 14F7-28mz CAR T cells inhibited tumor growth and significantly extended survival in both models (**Figures 3H, I**). No significant weight loss or other side effects were observed during daily monitoring over the course of the experiment. Bioluminescence imaging confirmed selective CAR T cell accumulation at the tumor site (**Figure 3J**). Together, these findings validate the efficacy and broad applicability of GM3(Neu5Gc) targeted CAR T cells, with apparent minimal toxicity to healthy tissues.

### Human 14F7 CAR T cells efficiently kill GM3(Neu5Gc)-expressing tumor cells *in vitro* and control established tumors

**Figure 4:**
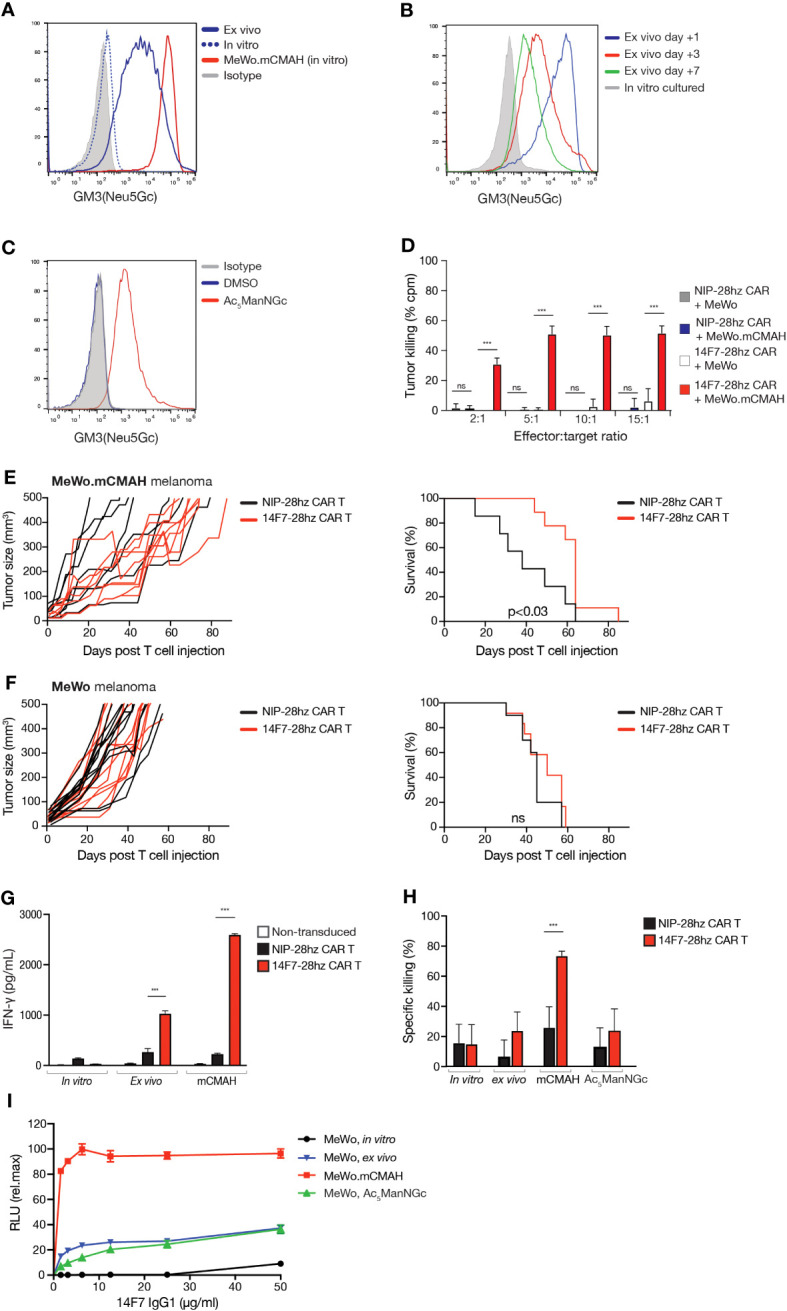
Human tumor cells can acquire GM3(Neu5Gc) surface expression and can be targeted by 14F7-28hz CAR T cells. **(A)** Histogram comparing GM3(Neu5Gc) expression by flow cytometry staining with 14F7 scFv of *in vitro* cultured, *ex vivo* isolated, and m*Cmah* expressing human melanoma MeWo cells. **(B)** Histogram showing flow cytometry staining with 14F7 scFv following *in vitro* propagation for several weeks of MeWo cells after *ex vivo* isolation. **(C)** Flow cytometry histogram showing staining with 14F7 scFv of MeWo cells metabolically labeled with 10 µM Ac_5_ManNGc (Vehicle=DMSO). **(D)** *In vitro* cytotoxicity of 14F7-28hz CAR T cells and irrelevant Nip-28hz CAR T cells incubated at the indicated effector-to-target ratios with MeWo and MeWo.m*Cmah* cells. **(E, F)** Tumor volume measurements and Kaplan-Meyer survival curve following adoptive transfer of 14F7-28hz or NIP-28hz CAR T cells in NSG mice harboring established (>5mm) subcutaneous MeWo.m*Cmah* (n=7-8) **(E)** and MeWo (n=12) **(F)** melanoma tumors. **(G)** IFN-γ secretion of 14F7-28hz and NIP-28hz CAR T cells co-cultured with m*Cmah*-expressing MeWo melanoma cells isolated from murine xenografts after tumor growth over several weeks. Data is presented as mean ± SD of one representative experiment of two. **(H)** Specific *in vitro* killing of 14F7-28hz compared to NIP-28hz CAR T cells co-cultured with *in vitro* cultured, *ex vivo* isolated, Ac_5_ManNGc-loaded and m*Cmah* expressing MeWo cells. Data is shown as mean ± SD from 6-8 replicates, in one representative experiment with three different PBMC donor **(I)** 14F7-IgG1 mediated antibody-dependent cellular cytotoxicity of *in vitro* cultured, *ex vivo* isolated, Ac_5_ManNGc-loaded and m*Cmah*-expressing MeWo cells. Shown is one representative experiment out of two, expressed as mean ± SD of technical replicates. RLU = relative light unit. *** p<0.001.

Numerous studies have reported strong 14F7 binding to human tumor cells in biopsy specimens (3–8). However, *in vitro* cultured human cancer cell lines typically show no detectable 14F7 antibody binding by flow cytometry (29), likely due to the absence of a functional *CMAH* gene in human cells. Despite the presence of Neu5Gc-containing antigens in bovine serum, no 14F7 immunostaining was observed in human tumor cells cultured in the presence of up to 30% FCS using either the murine 14F7 mAb or a humanized version of this antibody (14F7hT) (**Supplementary Figure 1C**). Consistent with previous reports (29), we found that introducing murine *Cmah* into the human MeWo tumor cell line and HEK-293T cells led to detectable GM3(Neu5Gc) expression under *in vitro* conditions (**Figure 4A**; **Supplementary Figure 2A**). Given the observed GM3(Neu5Gc) expression in murine tumor cells *in vivo*, we also assessed 14F7 mAb/scFv staining in human tumor xenografts, including MeWo melanoma, U87-MG glioma, and SKOV-3 ovarian adenocarcinoma cell lines. Most cell lines showed GM3(Neu5Gc) acquisition during *in vivo* growth, as determined *ex vivo* by flow cytometry and immunohistochemistry after allowing tumors to grow for several weeks (**Figure 4B**; **Supplementary Figure 2B, C** and **Supplementary Table 1**), aligning with prior reports (31, 32). However, GM3(Neu5Gc) expression levels did not match the expression levels of the MeWo.m*Cmah* variant and rapidly diminished when *ex vivo* isolated cells were cultured under standard *in vitro* conditions (**Figure 4B** and **Supplementary Figure 2B**).

Similar to observations in L1210.*Cmah*^-/-^ cells, GM3(Neu5Gc) surface expression could be induced in human tumor cell lines by adding the sialic acid precursor Ac_5_ManNGc to the culture medium (**Figure 4C** and **Supplementary Figures 2D, E**). This further underscores the ability of human tumor cells to incorporate Neu5Gc into their plasma membranes, even in the absence of a functional CMAH enzyme.

We subsequently developed a human 14F7-28hz CAR T cell. To verify its functionality, we co-cultured it with MeWo variants that displayed varying GM3(Neu5Gc) levels on their surfaces (**Figure 4A**). CD8^+^ 14F7-28hz CAR T cells eliminated MeWo.m*Cmah* cells but did not affect wild-type MeWo cells (**Figure 4D**). Considering that human tumor xenografts appear to acquire GM3(Neu5Gc) expression *in vivo*, we tested CAR T therapy by intravenous adoptive transfer in NSG mice with established subcutaneous MeWo xenografts measuring more than 5 mm in diameter. 14F7-28hz CAR T cells notably delayed tumor growth and extended the survival of mice with MeWo.m*Cmah* tumors (**Figure 4E**) but had no impact on mice challenged with wild-type MeWo cells (**Figure 4F**). Co-incubation of *ex vivo* isolated MeWo cells with 14F7-28hz CAR T cells triggered discernible CAR T signaling, albeit weaker than with MeWo.m*Cmah* cells (**Figure 4G**). However, the levels were not sufficient for tumor cell elimination (**Figure 4H**). In contrast, the addition of 14F7 mAb instigated significant antibody-dependent cell cytotoxicity (ADCC) against both *ex vivo*-isolated and metabolically labeled MeWo cells (**Figure 4I**). However, the activity was lower than with MeWo.m*Cmah* cells, suggesting that the lack of 14F7 CAR T cell killing stems from a limitation in the CAR format and not from an absence of 14F7-mediated recognition *per se.*

## Discussion

Carbohydrate antigens, while commonly used as diagnostic tumor markers, have yet to be fully harnessed for therapeutic purposes in immunotherapy. Among these, GM3(Neu5Gc) stands out due to its distinct expression profile, making it a promising candidate for precision immunotherapy.

Our findings underscore the capability of CAR T cells, which incorporate the antigen-binding domain of the 14F7 mAb, to efficiently and selectively target tumor cells expressing the GM3(Neu5Gc) ganglioside. The specificity of the 14F7 scFv is further corroborated by the loss of binding observed in *St3gal5* and *Cmah* knock-out cell lines. Notably, the adoptive transfer of 14F7-28mz CAR T cells in syngeneic mouse models led to the eradication of established solid tumors. Interestingly, some tumor cell lines, which lacked GM3(Neu5Gc) expression *in vitro*, showed accumulation of the ganglioside *in vivo*. This suggests that the ganglioside might be sourced either from an upregulated intrinsic *Cmah* expression or from the surrounding environment, as suggested by previous studies (20, 23, 24, 26). However, the absence of *in vivo* killing in human xenograft models hints that the latter mechanism might not achieve the GM3(Neu5Gc) expression levels required for effective elimination. Further studies using *Cmah*-deficient mice could provide clarity on the GM3(Neu5Gc) acquisition source in these scenarios.

A recent study by Cribioli et al. demonstrated that peritumoral injection of 14F7 CAR T cells delays the outgrowth of CMAH-expressing SKOV3 ovarian cancer xenografts injected four days earlier (28). While that paper utilized CARs based on mouse/human VH/VL 14F7 derived scFv chimeras sourced from the same publication as the murine VH/VL scFv (14F7c1) used in the present study, all scFv variants showed comparable binding affinities (10). Although indications of *in vitro* CAR T cell-mediated cytotoxicity against wild type SKOV3 cells was reported by Cribioli et al., the target-specific nature of these responses were not confirmed due to a lack of non-targeted CAR T cell controls (28). Similar to our results, no delay in tumor outgrowth was seen for wild-type human cancer cell xenografts, although the magnitude of GM3(Neu5Gc) acquisition during *in vivo* growth was not specifically addressed (28). We here substantiate and extend these findings, demonstrating that while GM3(Neu5Gc) expression on wild-type human tumor cells is attained during *in vivo* growth, and some level of 14F7 CAR T activation is induced, this is insufficient to elicit significant killing in established subcutaneous tumors.

Even though mice have a functional *Cmah* gene, the introduction of 14F7 CAR T cells did not lead to notable off-target side effects, suggesting that endogenous GM3(Neu5Gc) expression levels in healthy tissues were not sufficient for CAR T cell recognition and activation. Bioluminescence imaging of T cells further confirmed that 14F7-28mz CAR T cell accumulation was primarily located within the tumor site. This aligns with a recent study that observed no discernible side effects after adoptive transfer of human Neu5Gc-specific CAR T cells in NSG mice, albeit conducted in the absence of a tumor challenge (28).

Our human 14F7-28hz CAR T cells demonstrated the ability to recognize and eliminate human tumor cell lines that ectopically express murine CMAH, both *in vitro* and *in vivo*. However, human tumor cell lines with moderate GM3(Neu5Gc) expression remained resistant to CAR T-mediated elimination. Prior research indicates that dietary-derived Neu5Gc accumulates intracellularly over several months (33). Thus, the GM3(Neu5Gc) accumulation during human tumor development might be considerably more pronounced than what is observed during a xenograft experiment. While previous studies have indicated that the incorporation of Neu5Gc from FCS-containing medium in *in vitro* cultured human tumor cells (28), we failed to observe surface 14F7 immunostaining (either by humanized or murine mAb or scFv) in such cells even when passaged in a medium highly enriched with FCS. While the cause of these discrepancies remains uncertain, it is clear from our studies that Neu5Gc acquisition may occur during xenograft growth, suggesting that metabolic reprogramming or similar mechanisms may underlie this ability to incorporate Neu5Gc from the environment. Of note, previous reports have indicated that Neu5Gc incorporation from endogenous glycoconjugates involves the endosomal pathway (24), with subsequent cytosolic translocation via the sialic acid transporter SLC17A5, found to be preferentially expressed under hypoxic conditions (27). Alternatively, *de novo* synthesis, induced by hypoxia, has also been suggested as a possible mechanism of Neu5Gc acquisition (26).

The cytotoxic potential of 14F7 mAbs has been demonstrated in several studies (6, 16, 17, 29). In line with these findings, we find that human tumor cells grown *in vivo* acquire sufficient GM3(Neu5Gc) expression to elicit ADCC by 14F7 mAbs, albeit significantly less pronounced than CMAH-overexpressing tumor cells. Given the observed safety profile of 14F7 CAR T cells in *Cmah*-sufficient mice, and their limited efficacy against human tumor cells with low GM3(Neu5Gc) expression, we hypothesize that target affinity/avidity might be a limiting factor for 14F7 scFv-based therapies, especially in tumors with inconsistent antigen expression. Previous studies have used enzyme-linked immunosorbent assay (ELISA) to probe binding to immobilized gangliosides (10, 13), a system mimicking very high ganglioside density, but less representative of the cell membrane environment. In ELISA assays, both 14F7 (murine mAb) and various scFv formats performed similarly, showing low nanomolar affinities [*K*_d_ ≈ 15-40 nM (10); *K*_d_ ≈ 2-4 nM (13)] for GM3(Neu5Gc). Notably, this includes both the presently used 14F7c1 scFv (13) as well as the constructs employed in the recent study by Cribioli et al. (28).

Recent *in vitro* studies have highlighted differences in retention as well as a potential “all-or-nothing” threshold in 14F7 binding, contingent on the GM3(Neu5Gc) surface density (15). In that study, in contrast to previous reports, binding was assessed using ganglioside-containing liposomes, rather than gangliosides alone, and indicated *K*_d_ values of 3.4 μM for the scFv format *versus* 34 nM for the corresponding mAb, measured with electrochemiluminescence immunoassay (EIA) (15). The presence of two binding domains is expected to yield stronger retention of the mAb format compared to single antigen-binding domains when subjected to fluid dynamics, and might offer a potential explanation of the poor performance of 14F7 scFv-based CAR T cells against tumor cells with limited GM3(Neu5Gc) density.

The generally low affinity of naturally occurring antibodies against carbohydrate antigens remains a well-recognized challenge (34). To overcome this limitation in sensitivity towards low-abundance GM3(Neu5Gc), future strategies may require refined engineering of the kinetic profile, affinity, and CAR architecture. Alternatively, effective GM3(Neu5Gc)-directed CAR T cells may benefit from the incorporation of targeting elements derived from novel engineered antibodies or alternative scaffolding domains (35). Also, enhancing target availability in tumor cells through the delivery of exogenous Neu5Gc or synthetic sialic acid derivatives could be explored, as evidenced by the pronounced GM3(Neu5Gc) expression in human tumor cells exposed to the cell-permeable Neu5Gc precursor Ac_5_ManNGc *in vitro.* Successful *in vivo* glyco-engineering using various acetylated sialic acid precursors has been previously documented (36, 37).

The absence of detectable GM3(Neu5Gc) expression in *in vitro* cultured human cells poses challenges for the development and preclinical evaluation of therapies targeting this antigen. Our findings suggest caution when using murine cell lines or human cells with ectopically expressed functional *Cmah*, as the resulting expression levels might not mirror those in human tumors. Although the majority of tumor cell lines showed no detectable 14F7 mAb binding under standard *in vitro* conditions, consistent with prior reports (29), 14F7 mAb staining was consistently observed in *ex vivo* isolated tumor cells from established s.c. tumors of both murine and human origin. However, these expression levels rapidly diminished with continued *in vitro* propagation. The recent introduction of immunodeficient mice with a *Cmah* mutation mimicking that of humans (38) offers a promising avenue for evaluating novel GM3(Neu5Gc)-targeting therapies in xenograft models and gaining deeper insights into the selective accumulation of Neu5Gc in human cancer cells.

In summary, while there are evident limitations in 14F7 scFv binding to human tumor cells, our results bolster the potential of GM3(Neu5Gc) as a promising target for CAR T cell therapy across a diverse range of cancer subtypes.

## Methods

### Antibodies and flow cytometry

The structure and purification of the GM3(Neu5Gc)-specific 14F7 mAb (mouse IgG1 kappa) and a humanized version (14F7hT; human IgG1 kappa) have been previously described (3, 39). The single-chain variable fragment (scFv) 14F7c1 was derived by phage display light chain shuffling, and consists of the original murine 14F7 VH and a murine Ig kappa VL (10). Compared to the 14F7c1 scFv described by Bjerregaard-Andersen et al. (13), there are two substitutions in the VH domain, which correspond to corrections of the previously published sequence (PDB ID: 1RIH (14), corrected sequence in (40)). Gene fragments encoding the 14F7c1 scFv (13), an irrelevant 4-hydroxy-3-iodo-5-nitrophenylacetate (NIP) hapten-specific scFv (41), and corresponding anti-idiotypic scFvs (4G9 and 9A8, respectively) (42, 43) were constructed by *de novo* gene synthesis (Genscript Biotech Corp, NJ, USA) and inserted into the periplasmic expression vector pHOG (13). Expression and purification of scFv fragments were performed as previously described (44). Purified scFv fragments were biotinylated using EZ-Link Sulfo-NHS-Biotin (Thermo Fisher Scientific, MA, USA) according to the supplied manual. 14F7c1 expressed in a full-length mAb format was generated by transient transfection of Expi293 cells (ThermoFischer) using the pTRIOZ-hIgG1 expression vector system (*Invivo*Gen, Ca, USA). Flow cytometry was conducted using an Attune NtX Flow Cytometer (Thermo Fisher) and analyzed using FlowJo software v. 10 (FlowJo LLC, Ashland, OR, USA).

### Cell lines and *in vitro* culture experiments

All cell lines were derived from the American Type Culture Collection (ATCC, MA, USA) and cultured at 37°C in RPMI1640 GlutaMAX medium supplemented with 10% fetal bovine serum (FBS; Thermo Fisher), unless otherwise specified. For metabolic labeling, the sialic acid precursor *N*-glycolyl-d-mannosamine pentaacetate (Ac_5_ManNGc; TCI Europe, Zwijndrecht, Belgium) was dissolved in dimethyl sulfoxide (DMSO; Sigma-Aldrich, MO, USA), and 10 µM were added to standard culture medium. Tumor cells were grown in Ac_5_ManNGc-containing medium for 7 days to allow sufficient accumulation of GM3(Neu5Gc). Ac_5_ManNGc was replenished every second day.

### Mice and *in vivo* studies

C57BL/6J, BALB/c, BALB/c Rag2^-/-^, and immunodeficient NSG (NOD.Cg-Prkdc scid Il2rg tm1Wjl/SzJ) mice were obtained from Taconic labs and housed in a Minimal Disease Unit (MDU) at the Department of Comparative Medicine, Oslo University Hospital, Norway. Mice aged 6-8 weeks were utilized for *in vivo* tumor challenge experiments. For syngeneic tumor models, only female mice were utilized. In contrast, NSG xenograft experiments included both male and female mice, with equal gender distribution across the groups. Following tumor cell injection, mice were randomly assigned to different experimental groups. For L1210 and NS0 experiments in BALB/c Rag2^-/-^ mice, 1x10^6^ tumor cells suspended in PBS were administered by subcutaneous (s.c). injection. For B16 (C57BL/6J) and CT26 (BALB/c) experiments, 0.35x10^6^ B16 and 0.5x10^6^ CT26 tumor cells were administered intradermally (B16) or subcutaneously (CT26). After the s.c. tumors exceeded >5mm, immunocompetent C57BL/6J and BALB/c mice were preconditioned using a sublethal dose of X-ray irradiation (400 cGy) 24 hours prior to the adoptive T cell transfer of 4x10^6^ CAR T cells (1:1 mixed CD4^+^ and CD8^+^) administered by tail vein injection. For CAR T therapy experiments in immunodeficient mice, 4x10^6^ CAR T cells (1:1 mixed CD4^+^ and CD8^+^ CAR T cell populations) were injected without preconditioning. For human xenograft experiments, 1x10^6^ m*Cmah*-expressing MeWo cells were injected s.c. in 200 µl Matrigel (Corning Inc, NY, USA). Once the tumors were established, 8x10^6^ CAR T (1:1 mix of CD4^+^ and CD8^+^) cells were administered into the lateral tail vein. This was repeated 14 days later using 3x10^6^ CAR T cells. Mice were monitored daily, and tumor size was measured using a caliper. Tumor monitoring was not blinded. Luciferase-expressing CAR T cells were detected by bioluminescence imaging using the IVIS Spectrum *In Vivo* Imaging System (PerkinElmer, MA, USA), in accordance with a previously published protocol (45). Experiments were conducted according to institutional and governmental guidelines and were approved by the National Committee for Animal Experiments (Norwegian Food Safety Authority, Oslo, Norway), approval no. 13013/25037.

### Gene synthesis and viral transduction

The variable region amino acid sequence of an antibody against the hapten 4-hydroxy-3-iodo-5-nitrophenylacetic acid (NIP; clone B18) (41) was obtained from the RCSB Protein Data Bank (http://www.rcsb.org, PDB ID: 1A6U). DNA containing the coding sequence of the murine *Cmah* gene was purchased from OriGene (Cat. #MC219155; OriGene, MD, USA) and inserted into the 5´ open reading frame of the bicistronic retroviral expression vector MSCV-IRES-GFP (Addgene #20672; Addgene, MA, USA). Plasmid DNA encoding murine and human chimeric antigen receptors was constructed by *de novo* DNA synthesis (Genscript). To generate CAR T expression vectors, fragments encoding 14F7c1 or anti-NIP scFv sequences were fused to the CD28 transmembrane/signaling domain and CD3 zeta chain of murine and human origin, synthesized, and inserted downstream of a murine IL2 signal peptide sequence in the MSCV-IRES-GFP vector. For bioluminescence imaging, a previously generated MSCV-based vector expressing an enhanced version of firefly luciferase (MSCV-IRES-Effluc) was utilized (46). Retrovirus production and transduction were performed as previously described (47). For transduction of murine T cells, primary cells were obtained from splenocyte preparation by positive selection using anti-CD4 and anti-CD8 MACS beads (Miltenyi Biotec, Bergisch Gladbach, Germany). Human T cells were prepared from PBMCs isolated using Lymphoprep gradient centrifugation (Axis-Shield, Oslo, Norway) and selected using anti-CD4 and anti-CD8 MACS beads (Miltenyi Biotec). T cells were activated by a 48-hour incubation in anti-CD3/anti-CD28 mAb-coated cell culture plates and then transduced by a 30-minute centrifugation with retrovirus-containing supernatant in 24-well plates pre-coated with RetroNectin (Clontech Laboratories, CA, USA). After viral transduction, CAR T cells were expanded in RPMI+10% FBS and 100U/ml IL-2 (Peprotech, NJ, USA). To validate CAR transduction efficiency, transduced T cells were stained with biotinylated Protein L, 9A8 or 4G9 scFvs. Biotinylated scFv were detected using a PE conjugated streptavidin secondary antibody.

### CRISPR/Cas9 gene knock-out

Generation of CRISPR/Cas9 knock-out cell lines was performed as previously described (48). Briefly, the ablation of *Cmah*, *St3gal5*, and *B4galt1* genes was achieved by electroporating target cells using the Cas9/gRNA vector pSpCas9(BB)-2A-GFP (kindly provided by Dr. Feng Zhang via the Addgene repository; Addgene #48138) with the following guide RNA template sequences: m*Cmah*_s1: 5’-TAGTCGTACCCTCCAGGAAA-3’, m*St3gal5*_s1: 5’-TCGGGTGTACCATTGCAGGG-3’, and m*B4galt1*_s1: 5´-CAGGATGCGGCTAGACCGCC-3´. The presence of biallelic loss-of-function mutations in the resulting clones was confirmed by Sanger sequencing.

### Immunohistochemistry

Five µm-thick cryosections from paraffin-embedded tumor specimens were dewaxed, rehydrated, and washed in Tris-buffered saline (TBS; Sigma-Aldrich) solution for five minutes. Sections were blocked using a serum-free protein blocking solution (Agilent Technologies, CA, USA) for 10 minutes at room temperature. This was followed by incubation with 14F7 mAb (10 μg/mL) or biotinylated 14F7-scFv (20 μg/mL) in TBST buffer (20mM Tris-HCl, 150mM NaCl, 0.1% Tween 20, pH 7.4) for 1 hour in a humidified chamber. Negative control slides were incubated with equal concentrations of normal human IgG or an irrelevant scFv (NIP-scFv). After washing in TBS, sections were incubated for 30 minutes with the relevant detection agent (anti-human IgG-HRP/Streptavidin-HRP, Southern Biotech, Birmingham, AL, USA) in TBST buffer. HRP enzyme activity was detected using a diaminobenzidine tetrahydrochloride (DAB) colorimetric substrate (Thermo Fischer). Sections were counterstained with Mayer’s hematoxylin (Sigma-Aldrich) and mounted with an aqueous mounting medium.

### Cytotoxicity and cytokine release assays

For cytotoxicity assays, target cells were labeled with methyl-[3H] thymidine (3H-TdR, Montebello Diagnostics, Oslo, Norway) 16 hours prior at 37°C. 1x10^4^ target cells were seeded in each well of a 96-well plate. CD8^+^ CAR T cells were added at the indicated effector-to-target (E:T) ratios. After 24 hours of co-culture, plates were harvested and analyzed on a TopCount NXT microplate counter (Perkin Elmer, Shelton, CT). For cytokine release assays, CD8^+^ CAR T cells were incubated with target cells at a 1:2 ratio. After 24 hours, the conditioned culture medium was collected and analyzed using Human IFN-γ, human IL-2, or murine IL-2 DuoSet ELISA kits (R&D Systems, Minneapolis, MN, USA).

### Antibody-dependent cellular cytotoxicity assay

Jurkat-Lucia™ NFAT-CD16 reporter cells was obtained from *In vivo*Gen (*In vivo*Gen, CA, USA) and maintained in RMPI supplemented with 10% FBS, 10 μg/ml blasticidin and 100 μg/ml zeocin. ADCC reporter assay was performed according to the manufacturer’s instructions. In brief, 1x10^5^ tumor cells were incubated with the indicated amount 14F7-IgG1 antibody for 1h at 37°C in a 96-well plate. As a negative control polyclonal human IgG1 antibody (BioXcell, NH, USA) was used. Next, 2x10^5^ Jurkat-Lucia™ NFAT-CD16 were added, and plates was incubated at 37°C. After 18h incubation, QUANTI-Luc™ 4 Reagent (*In vivo*Gen) was added to the plate and immediately read out on a GloMax 96 Microplate Luminometer (Promega, WI, USA). RLU of max was calculated relative to the highest concentration (50 μg/ml) of 14F7-IgG1 antibody.

### Statistical analysis

Cytotoxicity assay, cytokine release assay and ADCC assay data were analyzed using one-way ANOVA. *In vivo* bioluminescent data was analyzed using a non- parametric Mann-Whitney test. Survival data of mice treated with either Nip-28z CAR T cells or 14F7-28z CAR T cells were compared using the Mantel-Cox Log-Rank test. All statistical analyses were performed using the GraphPad Prism software (GraphPad Software, CA, USA, version 9.3.1). P-values were considered statistically significant: * p<0.05; ** p<0.01; *** p<0.001; ns= not significant.

## Data availability statement

The raw data supporting the conclusions of this article will be made available by the authors, without undue reservation.

## Ethics statement

Ethical approval was not required for the studies on humans in accordance with the local legislation and institutional requirements because only commercially available established cell lines were used. The animal study was approved by Norwegian National Committee for Animal Experiments (Norwegian Food Safety Authority, Oslo, Norway). The study was conducted in accordance with the local legislation and institutional requirements.

## Author contributions

JH: Data curation, Formal analysis, Investigation, Methodology, Writing – original draft, Writing – review & editing. MF: Conceptualization, Data curation, Formal analysis, Supervision, Writing – review & editing. IJ: Conceptualization, Data curation, Formal analysis, Supervision, Writing – review & editing. UK: Conceptualization, Formal analysis, Methodology, Project administration, Resources, Supervision, Writing – review & editing. GL: Conceptualization, Formal analysis, Methodology, Resources, Supervision, Writing – review & editing. LM: Formal analysis, Funding acquisition, Methodology, Resources, Supervision, Writing – original draft, Writing – review & editing. AT: Conceptualization, Data curation, Formal analysis, Funding acquisition, Methodology, Project administration, Supervision, Writing – original draft, Writing – review & editing.
