# Supplementary material for: Chimeric antigen receptor T cells targeting the GM3(Neu5Gc) ganglioside
Source: Front Immunol. 2024 Feb 2;15:1331345. doi: 10.3389/fimmu.2024.1331345 (PMC10869436; doi:10.3389/fimmu.2024.1331345)
Supplement: Supplementary file 1 [file DataSheet_1.docx]

**Supplementary Figure 1**


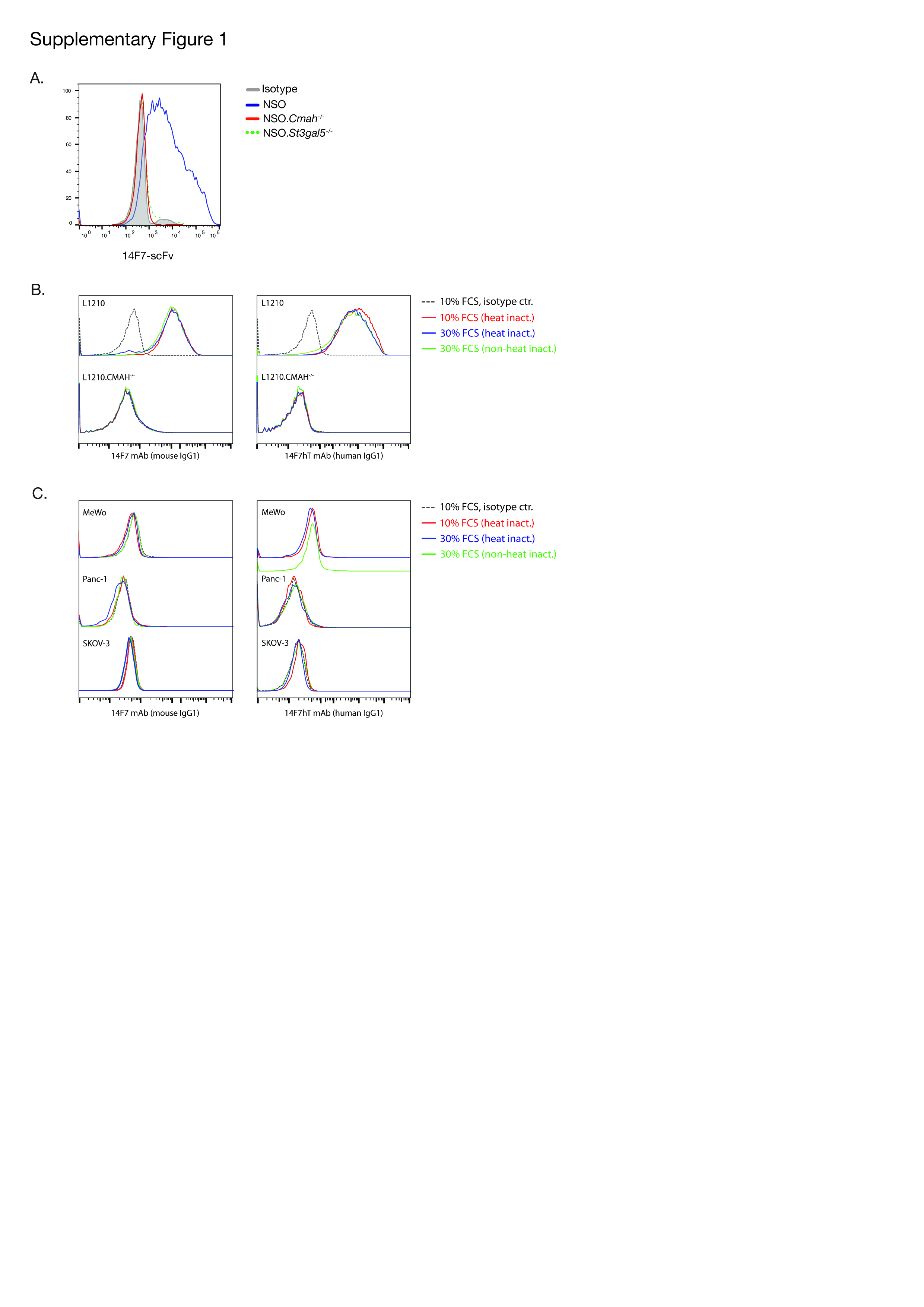


**(A)** Representative flow cytometry histogram showing 14F7 scFv staining of wild type (WT) NS0, NS0.cmah^-/^, and NS0.st3gal5^-/-^cells. **(B-C)** Representative flow cytometry histograms showing staining of murine (**B**) and human (**C**) cell lines with murine (left panel) and humanized (right panel; 14F7hT) versions of the 14F7 mAb. Prior to staining, cells were passaged and cultured at 37C in 5% Co2 atmosphere in medium containing heat-inactivated or non-heat-inactivated fetal calf serum (FCS) at the indicated %Vol. Dotted line shows staining with a corresponding isotype-matched control mAb.

**Supplementary Figure 2**


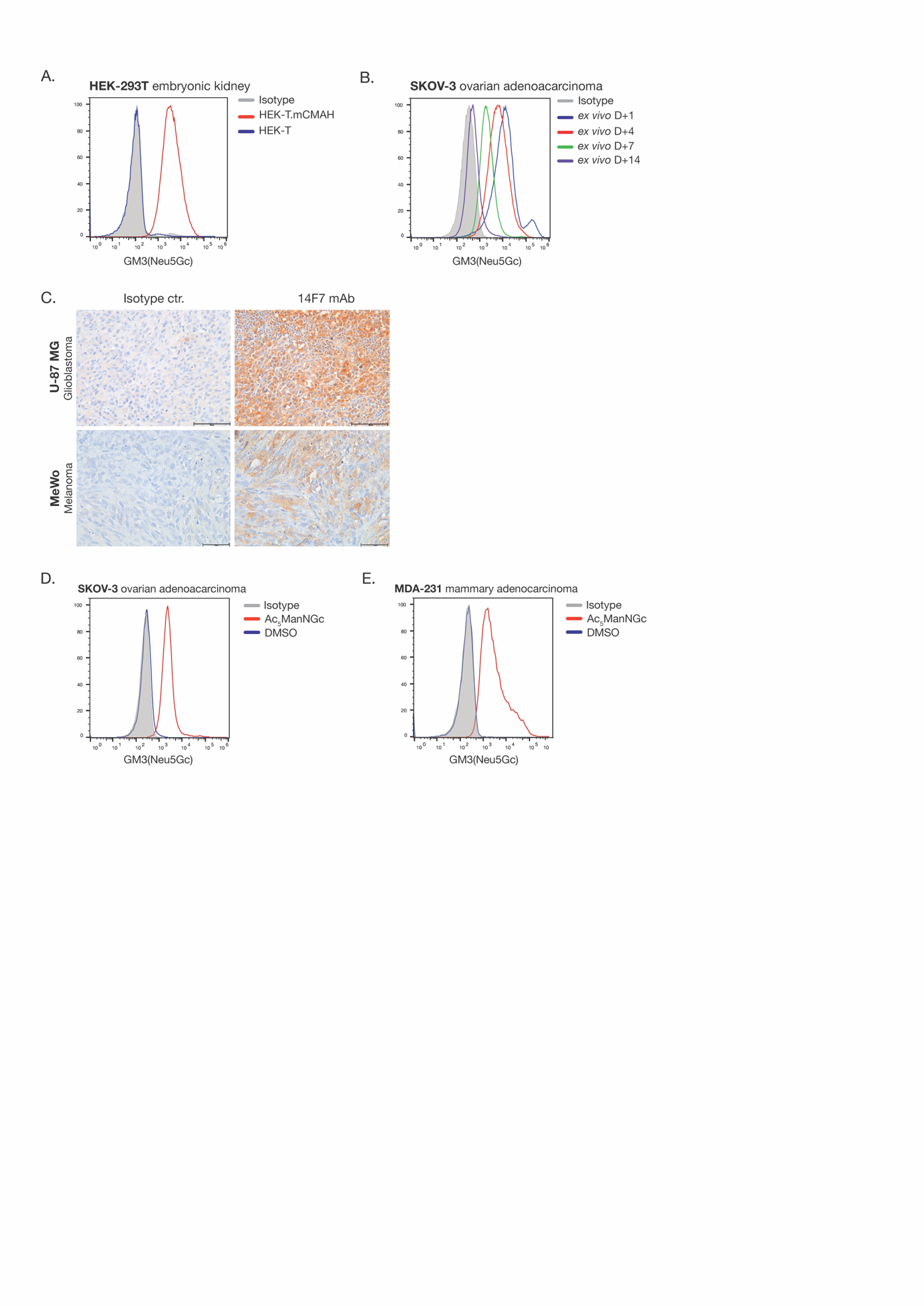


**(A)** Histogram comparing flow cytometry staining of WT and m*Cmah* expressing HEK-293T cells. **(B)** Histogram comparing flow cytometry staining of *ex vivo* isolated SKOV-3 cells propagated *in vitro*. **(C)** Immunohistochemistry staining with 14F7 mAb of large established (> 5 mm) subcutaneous U-87 MG glioblastoma and MeWo melanoma tumors. Histogram comparing flow cytometry staining of **(D)** SKOV-3 and **(E)** MDA-231 cells metabolically labeled with Ac_5_ManNGc.

**Supplementary Table 1**

| **Cell line** | **Species** | **Origin** | **14F7 mAb surface staining** | | | |
| --- | --- | --- | --- | --- | --- | --- |
|  |  |  | In vitro (FC) | Ex vivo (FC) | | Ex vivo (IHC) |
| A20 | Mouse | *Lymphoma* | - | | + | + |
| B16 | Mouse | *Melanoma* | - | | + | + |
| MOPC315 | Mouse | *Plasmacytoma* | - | | + | + |
| CT26 | Mouse | *Colon adenocarcinoma* | - | | + | + |
| X63 | Mouse | *Plasmacytoma* | + | | + | + |
| NS0 | Mouse | *Plasmacytoma* | + | | + | + |
| L1210 | Mouse | *Lung adenocarcinoma* | + | | + | + |
|  |  |  |  | |  |  |
| MeWo | Human | *Melanoma* | - | | + | + |
| SKOV-3 | Human | *Ovarian adenocarcinoma* | - | | + | + |
| L428 | Human | *Hodgkin lymphoma* | - | | N/A | N/A |
| Mino | Human | *Mantle Cell Lymphoma* | - | | N/A | N/A |
| Ramos | Human | *Burkitt's lymphoma (EBV negative)* | - | | - | N/A |
| Raji | Human | *Burkitt's lymphoma (EBV positive)* | - | | - | N/A |
| K422 | Human | *Diffuse large B cell lymphoma* | - | | N/A | N/A |
| SUDHL-6 | Human | *Diffuse large B cell lymphoma* | - | | N/A | N/A |
| U-2932 | Human | *Diffuse large B cell lymphoma* | - | | N/A | N/A |
| U-2940 | Human | *Diffuse large B cell lymphoma* | - | | N/A | N/A |
| Jurkat | Human | *T cell leukemia* | - | | N/A | N/A |
| RPMI 8266 | Human | *B-cell Non-Hodgkin's lymphoma* | - | | - | N/A |
| HDLM-2 | Human | *Hodgkin lymphoma* | - | | N/A | N/A |
| Panc-1 | Human | *Pancreatic adenocarcinoma* | - | | - | N/A |
| BxPC-3 | Human | *Pancreatic adenocarcinoma* | - | | (+) | N/A |
| U-87 | Human | *Glioblastoma* | - | | + | + |

Table showing results of surface flow cytometry (FC) and immunohistochemistry (IHC) staining of murine and human cancer cell lines using 14F7 mAb/scFv. Positive staining was determined subjectively based on comparison to staining with an isotype control mAb.
N/A – not applicable.
